# Therapeutic Efficacy and Safety of Paclitaxel/Lonidamine Loaded EGFR-Targeted Nanoparticles for the Treatment of Multi-Drug Resistant Cancer

**DOI:** 10.1371/journal.pone.0024075

**Published:** 2011-09-08

**Authors:** Lara Milane, Zhenfeng Duan, Mansoor Amiji

**Affiliations:** 1 Department of Pharmaceutical Sciences, School of Pharmacy, Northeastern University, Boston, Massachusetts, United States of America; 2 Department of Orthopaedic Surgery, Massachusetts General Hospital, Boston, Massachusetts, United States of America; 3 Sarcoma Biology Laboratory, Center for Sarcoma and Connective Tissue Oncology, Massachusetts General Hospital, Boston, Massachusetts, United States of America; Aristotle University of Thessaloniki, Greece

## Abstract

The treatment of multi-drug resistant (MDR) cancer is a clinical challenge. Many MDR cells over-express epidermal growth factor receptor (EGFR). We exploit this expression through the development of EGFR-targeted, polymer blend nanocarriers for the treatment of MDR cancer using paclitaxel (a common chemotherapeutic agent) and lonidamine (an experimental drug; mitochondrial hexokinase 2 inhibitor). An orthotopic model of MDR human breast cancer was developed in nude mice and used to evaluate the safety and efficacy of nanoparticle treatment. The efficacy parameters included tumor volume measurements from day 0 through 28 days post-treatment, terminal tumor weight measurements, tumor density and morphology assessment through hematoxylin and eosin staining of excised tumors, and immunohistochemistry of tumor sections for MDR protein markers (P-glycoprotein, Hypoxia Inducible Factor, EGFR, Hexokinase 2, and Stem Cell Factor). Toxicity was assessed by tracking changes in animal body weight from day 0 through 28 days post-treatment, by measuring plasma levels of the liver enzymes ALT (Alanine Aminotransferase) and LDH (lactate dehydrogenase), and by white blood cell and platelet counts. In these studies, this nanocarrier system demonstrated superior efficacy relative to combination (paclitaxel/lonidamine) drug solution and single agent treatments in nanoparticle and solution form. The combination nanoparticles were the only treatment group that decreased tumor volume, sustaining this decrease until the 28 day time point. In addition, treatment with the EGFR-targeted lonidamine/paclitaxel nanoparticles decreased tumor density and altered the MDR phenotype of the tumor xenografts. These EGFR-targeted combination nanoparticles were considerably less toxic than solution treatments. Due to the flexible design and simple conjugation chemistry, this nanocarrier system could be used as a platform for the development of other MDR cancer therapies; the use of this system for EGFR-targeted, combination paclitaxel/lonidamine therapy is an advance in personalized medicine.

## Introduction

The development of **multi-drug resistant (MDR) cancer** often impedes the clinical treatment of cancer as it results in non-responsive disease that can lead to metastasis [1], [2]. MDR refers to a state of resilience against structurally and/or functionally unrelated drugs [1]. MDR is often acquired through exposure to chemotherapeutic agents but MDR can also be intrinsic (innate) [1].

Hypoxia is an established microenvironmental selection pressure that can result in MDR and resistance to radiation therapy [3], [4]. Under conditions of hypoxia and cell stress Hypoxia Inducible Factor alpha (HIF-1α) translocates from the cytoplasm to the nucleus; HIF-α then complexes with HIF-β, forming an active transcription factor [3], [4]. This active HIF complex is then able to induce transcription by binding to Hypoxia Responsive Elements (HRE's) on target genes; target genes include P-glycoprotein (P-gp), Epidermal Growth Factor Receptor (EGFR), and many glycolytic proteins such as Hexokinase 2 (HXK2) [3], [4]. Oxygen-independent factors such as cyclooxygenase-2 activity, epidermal growth factor receptor (EGFR), heat-shock protein 90, and phosphatidylinositol 3-kinase can also stabilize HIF [4], [5], [6].

P-gp is a transmembrane drug efflux pump of the ATP-Binding Cassette (ABC) transporter family; P-gp expression in cancer is associated with MDR and poor prognosis [2]. EGFR expression in some types of cancer is also associated with aggressive disease [7]. Over expression of EGFR leads to receptor clustering in the cell membrane which makes a cell hyper-sensitive to EGFR substrates; this aids the survival of MDR cells, especially hypoxic tumor regions that may be distal from a continuous nutrient supply [7].

Another survival advantage for cancer cells is to acquire energy through glycolysis; either anaerobic (the Pasteur Effect) or aerobic (the Warburg Effect) [8]. Many glycolytic proteins such as hexokinase 2 are HIF targets [3], [4], [9]. Hexokinase catalyzes the first step of glycolysis; the hexokinase 2 isoform is directly associated with mitochondria and is overexpressed in many types of cancer [4], [10], [11]. Mitochondrial association of hexokinase 2 prevents binding of pro-apoptotic BcL-2 family member proteins through spatial inhibition of the mitochondrial permeability transition pore complex; this also aids cell survival as it prevents cytochrome c release and the subsequent apoptotic cascade [10].

The current drug delivery system actively targets MDR cancer cells through EGFR binding; the surface of the nanocarriers have been modified with an EGFR-specific peptide. This system treats MDR cancer by using a combination of paclitaxel and lonidamine. Paclitaxel (PTX) is a common chemotherapeutic agent that hyper-stabilizes microtubules, preventing cell division; PTX is a non-specific agent and is associated with high residual toxicity. Lonidamine (LON) (1-[(2,4-Dichlorophenyl)methyl]-1H-indazole-3-carboxylic acid) is a hexokinase 2 inhibitor that has been shown to induce apoptosis and treat MDR in various cancer cell lines [12], [13], [14]. In the United States, Phase II clinical trials of LON as a treatment for benign prostatic hyperplasia were suspended due to associated liver toxicity [15], [16]. This drug delivery system aims to improve the efficacy and reduce the toxicity of PTX and LON through the use of combination therapy and active targeting.

**Figure 1:**
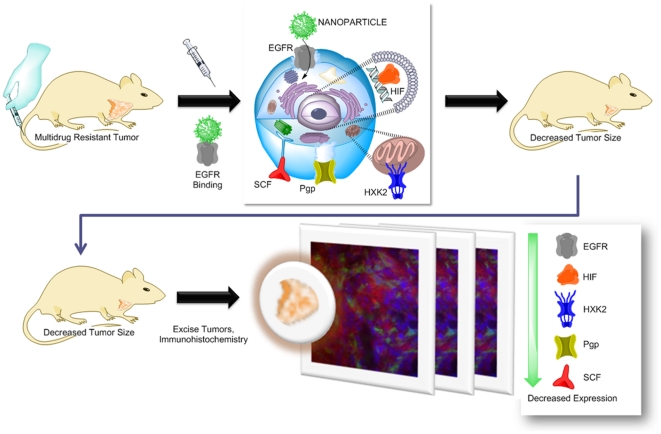
Treatment schema. Nude mice with orthotopic, multidrug resistant breast tumor xenografts were treated with EGFR-targeted, polymer blend nanoparticles loaded with paclitaxel and lonidamine. This nanoparticle formulation is internalized via the EGFR receptor; treatment leads to a cascade of cellular changes and a decrease in tumor volume. As assessed by immunohistochemistry of tumor xenografts, nanoparticle treatment decreased the expression of hypoxic and MDR markers (EGFR, epidermal growth factor receptor; HIF, hypoxia inducible factor; HXK2, hexokinase 2; Pgp, P-glycoprotein; SCF, stem cell factor).

This study examines the therapeutic efficacy and safety of EGFR-targeted nanoparticles (NPs) loaded with PTX and LON. These polymer-blend nanocarriers were evaluated in an orthotopic model of MDR breast cancer. Tumor size and growth progression was used to assess the efficacy of therapy. The safety/toxicity of this therapy was assessed by measuring the change in body weight, plasma levels of the liver enzymes ALT (Alanine Aminotransferase) and LDH (lactate dehydrogenase), and white blood cell and platelet counts. To further characterize the efficacy of this therapy, H & E staining of tumor sections from each group were compared. Also, Immunohistochemistry (IHC) of the tumor sections for expression of P-gp, HIF-1α, EGFR, HXK2, CD-31, and Stem Cell Factor (SCF) was used to assess the MDR character of the tumors after treatment. As demonstrated by the schema in **Figure 1**, treatment with the EGFR-targeted NPs loaded with PTX and LON decreased tumor volume and decreased the expression of hypoxic and MDR associated proteins in the orthotopic breast cancer model.

## Results

### Nanoparticle Characterization

**Figure 2:**
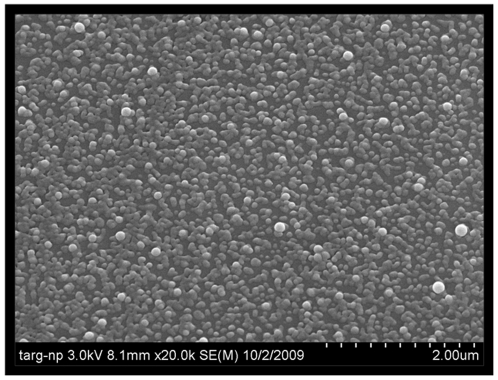
SEM of nanoparticles. The nanometer size of the nanocarriers was confirmed with a scanning electron micrograph of the nanoparticles. The scale bar is 2 µm.

The design objective of this nanocarrier system was to actively target MDR cells by binding to the EGFR receptor and subsequently deliver PTX and LON to the site of a tumor. To achieve appreciable loading efficiency for both drugs (approximately ≥70%) PCL (Polycaprolactone) was used as the primary nanoparticle constituent (formulation optimization is described in our previously published work [17]). To achieve active targeting a PLGA-PEG-Peptide construct was synthesized and incorporated in the PCL (Polycaprolactone) NPs. Both a PLGA-PEG and a PLGA-PEG-Peptide construct were incorporated in the formulation to achieve surface modification with PEG and the peptide. The PLGA of the construct interacts with the PCL core of the particles, aiding in lonidamine and paclitaxel encapsulation while the PEG and EGFR-specific peptide protrude from the particle surface enabling active targeting and protection from the reticuloendothelial system. The complete synthesis and characterization of this system is described in our other work [17]; NMR was used to assess the grafting process, ESCA was used to confirm the presence of the peptide on the surface of the NPs, drug encapsulation and release kinetics were quantified over time, and EGFR targeting was quantified in a panel of nine cell lines with various levels of EGFR expression. As depicted in **Figure 2**, SEM of the NPs confirmed the nanometer scale of the particles which averaged between 120 –160 nm.

### Efficacy Analysis

MDA-MB-231 tumors established from hypoxic pre-conditioned cells were grown to 100 mm^3^ size and then mice were treated with one of the following 8 treatments; (1) targeted NPs loaded with PTX and LON, (2) SOL of PTX and LON, (3) targeted NPs loaded with PTX, (4) PTX SOL, (5) targeted NPs loaded with LON, (6) LON SOL, (7) blank targeted NPs (no drug), and (8) saline. Treatment proceeded for 28 days. During this time, tumor size and body weight were monitored and blood was collected to assess toxicity.

**Figure 3:**
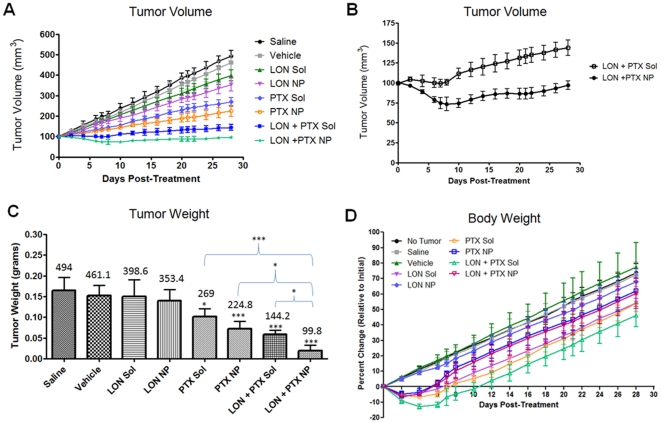
Tumor volume, tumor weight, and body weight after treatment. Efficacy of the combination nanoparticle therapy was assessed by measuring changes in tumor volume from 0–28 days post-treatment and by measuring the terminal tumor weight at 28 days post-treatment. Day 0 was the first day of treatment. Body weight was used as a toxicity parameter and was measured from 0–28 days post-treatment. For all graphs, each data point represents the mean ± SD with an *n* = 4. (**A**) Tumor volume. Mice were treated with one of eight treatments via tail vein injection; saline (black circles), vehicle (blank nanoparticles; gray squares), lonidamine solution (LON Sol; green triangles), lonidamine nanoparticles (LON NP; purple inverted triangles); paclitaxel solution (PTX Sol; light blue diamonds), paclitaxel nanoparticles (PTX NP; orange open circles), lonidamine and paclitaxel combination solution (LON + PTX Sol; dark blue asterisks), and lonidamine and paclitaxel combination nanoparticles (LON + PTX NP; light green stars). (**B**) Tumor volume (of combination therapy). To clarify the difference between lonidamine and paclitaxel combination treatment in solution form (LON + PTX Sol; open circles) compared to nanoparticle treatment (LON + PTX NP; closed circles), these two groups were graphed separately here. (**C**) Tumor weight. The terminal tumor weight (at 28-days post-treatment) was plotted for each treatment group. The number above each bar represents the average terminal tumor volume (from panel A). The asterisks directly above the error bars indicate significance between saline and the indicated group (*p<0.05, ***p<0.001). (**D**) Body weight. Body weight was measured and plotted as percent change (relative to initial body weight) from 0–28 days post-treatment. The groups include; no tumor (black circles), saline (gray squares), vehicle (green triangles), lonidamine solution (LON Sol; purple inverted triangles), lonidamine nanoparticles (LON NP; light blue diamonds), paclitaxel solution (PTX Sol; orange open circles); paclitaxel nanoparticles (dark blue open squares), lonidamine and paclitaxel combination solution (LON + PTX Sol; light green open triangles), and lonidamine and paclitaxel combination nanoparticles (LON + PTX NP; pink inverted open triangles).

**Figure 3** **.A** depicts the tumor growth in each treatment group from day 0 (date of treatment initiation) until day 28. The tumor growth for the saline and vehicle (blank NP) treated groups is similar; while treatment with LON SOL and LON NPs resulted in slightly decreased tumor volume. Treatment with PTX SOL and PTX NPs resulted in a further decrease in tumor volume. Treatment with combination (LON and PTX) SOL actually repressed tumor growth for 10 days, at which point growth resumed at a much slower rate. This is illustrated in **Figure 3** **.B** along with the combination NP treatment. The combination NPs were the only treatment group that actually decreased tumor volume, sustaining this decrease until the 28 day time point when the tumor volume approached the initial tumor volume.

A Two-way ANOVA test was used to analyze the data; the results are presented in **Table 1**. There was no significance for any time point between the saline and vehicle treated groups. For this reason, comparisons of each group with the vehicle treatment group were not included in **Table 1**. **Table 1** compares the treatment groups and lists the day post-treatment that a specific level of significance was reached. Most notably, between 4 and 12 days, there is significance between both the combination SOL group and the combination NP group compared to saline, LON SOL, LON NPs, PTX SOL, and PTX NP treatments. There is also significance between the combination SOL group and the combination NP group after 10 days of treatment.

**Table 1:** Two-Way ANOVA of tumor growth.

Comparison		Significance Level,Days Post-Treatment		
Treatment A	Treatment B	*p*<0.05	*p*<0.01	*p*<0.001
Saline	LON SOL	---	14	18
Saline	LON NP	---	10	12
Saline	PTX SOL	---	---	6
Saline	PTX NP	4	---	6
Saline	LON + PTX SOL	---	---	4
Saline	LON + PTX NP	---	---	4
LON SOL	LON NP	22	---	---
LON SOL	PTX SOL	8	---	10
LON SOL	PTX NP	6	7	8
LON SOL	LON + PTX SOL	4	---	6
LON SOL	LON + PTX NP	---	---	4
LON NP	PTX SOL	14	16	20
LON NP	PTX NP	8	10	14
LON NP	LON + PTX SOL	---	---	6
LON NP	LON + PTX NP	---	4	6
PTX SOL	PTX NP	18	21	---
PTX SOL	LON + PTX SOL	7	8	12
PTX SOL	LON + PTX NP	---	---	6
PTX NP	LON + PTX SOL	12	14	18
PTX NP	LON + PTX NP	---	6	7
LON + PTX SOL	LON + PTX NP	10	20	---

GraphPad Prism® Software was used to perform a two-way ANOVA of the tumor growth data after treatment (this data is graphed as Tumor Volume from 0-28 days post-treatment in Figure 3.A). Treatment A (first column) was compared to treatment B (second column) and the time is took (in days-post-treatment) to reach a significance level of p<0.05, p<0.01, and p<0.001 are listed in columns three, four, and five respectively.

The tumor weights from each group are presented in **Figure 3** **.C**. The mean tumor volume on day 28 (sacrifice) is indicated above the bar for each group. The tumor weights correspond with the terminal tumor volume data. Combination therapy with EGFR-targeted NPs was significantly more effective at reducing tumor volume than single agent treatment.

### Safety and Toxicity Profiles

The body weight of each group was also monitored throughout the course of treatment and is presented in **Figure 3** **.D**. All SOL groups underwent an initial decrease in body weight, which remained lower throughout the course of treatment. This effect was the most pronounced for the combination SOL. The groups treated with PTX NPs and with the combination NPs also underwent an initial decline in body weight; however, there was a moderate recovery of this decline between 7-10 days post-treatment. The initial and sustained decline of the SOL groups is most likely due to the Cremophor® EL solution. The decline in body weight associated with the combination NPs and the PTX NPs is most likely due to the cytotoxicity of PTX.

**Figure 4:**
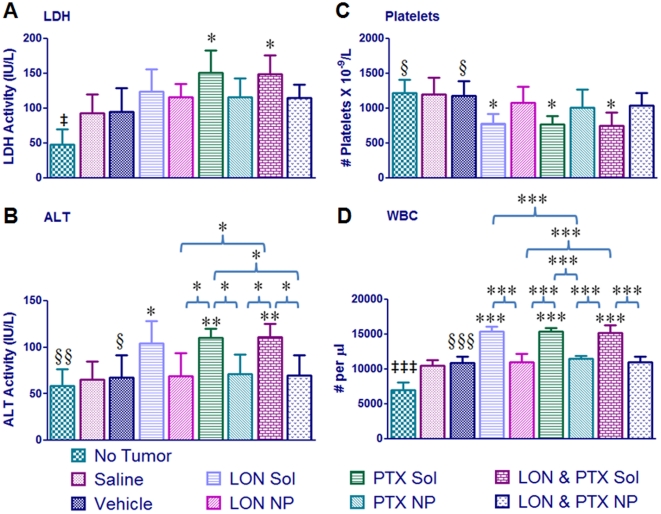
Toxicity analysis. Liver enzymes and blood counts were used to assess toxicity. For all graphs, each data point represents the mean ± SD with an *n* = 4. The double dagger represents significance between the No Tumor group and all other groups (‡ *p*<0.05, ‡‡ *p*<0.01, ‡‡‡ *p*<0.0001). The section sign represents significance between the indicated group and all solution groups (§ *p*<0.05, §§ *p*<0.01, §§§ *p*<0.0001). The asterisks directly above the bars (no brackets) represents significance between that group and the saline group whereas asterisks above brackets indicate inter-group significance (*p<0.05, **p<0.01, ***p<0.0001). (**A**) Lactate dehydrogenase (LDH). (**B**) Alanine Aminotransferase (ALT). (**C**) Platelet Counts. (**D**) White Blood Cell (WBC) Counts.

Blood samples were also collected on day 0, day 14, and day 28 of treatment and were analyzed for levels of LDH and ALT as well as white blood cell and platelet counts. The results of the day 14 blood analysis are portrayed in **Figure 4**; for all four parameters, there was no significance between any treatment group at day 0. LDH (**Figure 4** **.A**) is often elevated in cancer and due to tissue damage, as such; it is a common marker for disease and toxicity. At day 14 there was significance between the saline group and the group treated with PTX SOL as well as the group treated with combination SOL. There was significance between the No Tumor group and all treatment groups at day 14 (p<0.01) and at day 28 (p<0.05; **Figure S1**). This is most likely due to the growth of the tumor itself, and not directly associated with the treatment administered to each group. An increase in ALT (**Figure 4** **.B**) is often associated with liver damage. At day 14, there was significance between the saline group and all SOL groups. Of importance, at this time point there was also significance between the combination SOL and the combination NPs. Combination LON/PTX therapy with NPs results in less liver toxicity than treatment with combination SOL. There was also significance between the No Tumor group (and the Vehicle group) and all SOL treatment groups at day 14 (*p*<0.01 for No Tumor comparison, *p*<0.05 for Vehicle comparison).

A decrease in platelet counts (**Figure 4** **.C**) is often associated with chemotherapy toxicity. At day 14, there was significance between the No Tumor group and all SOL groups. At this time point there was also significance between the saline (and vehicle) group and all SOL groups. Elevated WBC counts (**Figure 4** **.D**) are indicative of tissue damage. Consistent with the other toxicity data, there is significance between the saline and vehicle treated groups and all of the SOL treated groups. There is also significance between each SOL group and the corresponding NP group. The NP treatments are less toxic than SOL drug treatments. There is significance between the No Tumor group and all other groups. As this difference is apparent in the saline and vehicle groups also, it is most likely an indication of tissue damage associated with tumor development in the mice. Collectively, the blood analysis indicates that NP therapy is significantly less toxic than SOL drug treatment.

### Histology

**Figure 5:**
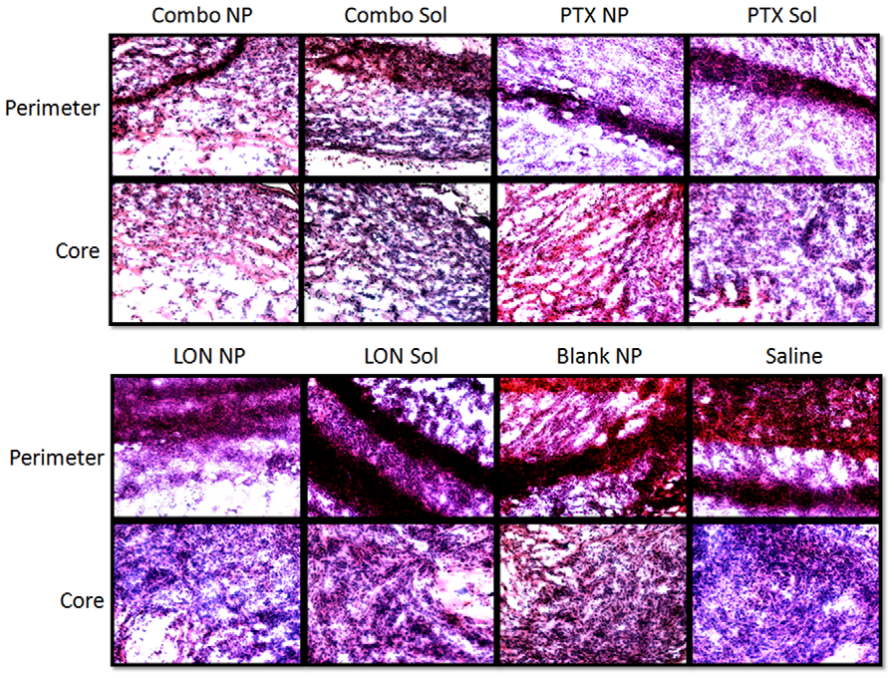
Histology of tumor perimeters and cores. Tissue sections were stained with hematoxylin and eosin. The nuclei are stained with hematoxylin (blueish color) while other cell fractions are stained with eosin (red and pink colors).

To further assess toxicity, hematoxylin and eosin staining of tumor sections from each treatment group were examined (**Figure 5**). Images of both the tumor perimeter and the tumor core were acquired. Common to all tumor sections, is the haphazard array of cell growth. Each tumor has a clear demarcation of increased cell density in the perimeter of the tumor. This perimeter, however, is not consistent between the treatment groups. The saline treated group, the group treated with blank NPs, and the group treated with LON SOL appear to have a much thicker tumor boundary of cells. This appears slightly decreased in the LON NP treated group, and more markedly decreased in the groups treated with PTX SOL, PTX NPs, combination SOL, and combination NPs. The core density appears to follow a similar pattern as the perimeter density in the treatment groups. The cell density in the tumor core sections of the saline group, the blank NP group, the LON SOL group, and the LON NP group seem much higher than the cell densities of the PTX SOL group, the PTX NP group, the combination SOL group, and the combination NP group. The decreased cell density of the combination NP group may explain the dramatic decrease in the final tumor weight. These decreases in density may be a hallmark of effective treatment.

**Figure 6:**
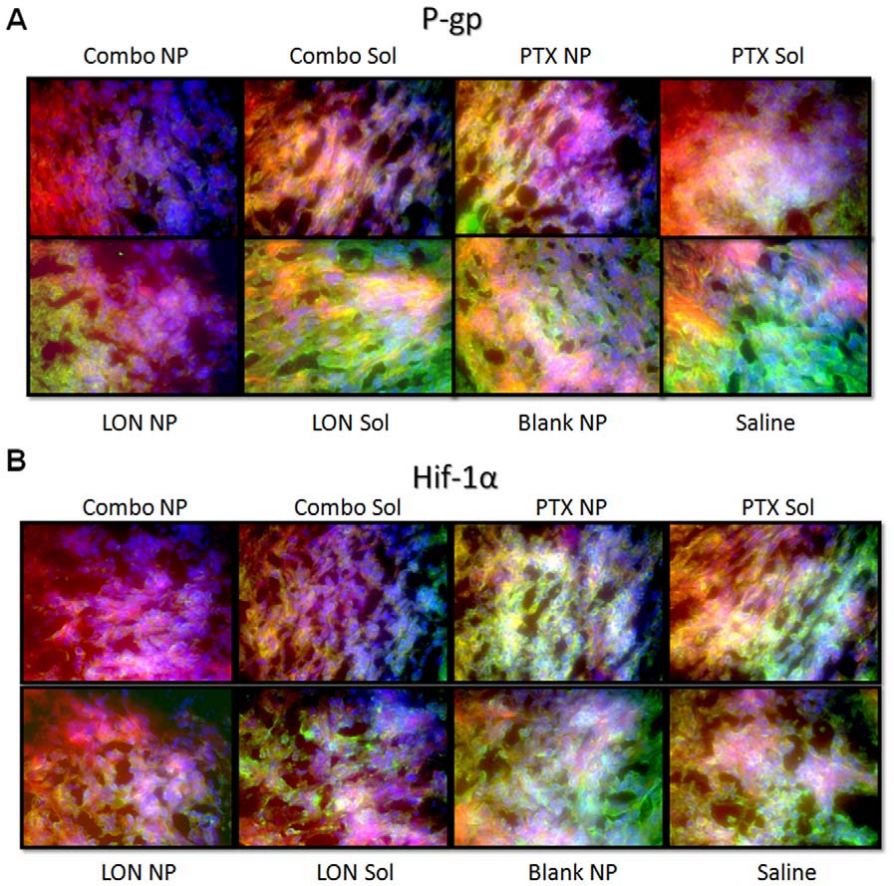
Immunohistochemistry of P-gp and HIF-1α. Tissue sections were probed with primary antibodies against the protein of interest, then labeled with Alexa Fluor® 488 conjugated secondary antibodies (green). F-actin was stained with Alexa Fluor® 568 phalloidin (red) and nuclei were stained with Hoechst 33342 (blue).

IHC of tumor section from each group were analyzed for expression the following six proteins; P-gp, HIF-1α, EGFR, CD-31, HXK2, and SCF (**Figure 6** **–** [Fig pone-0024075-g007] **8**). For each section, F-actin is stained with phalloidin (red), nuclei are stained with Hoechst 33342 (blue), and the protein of interest is labeled with secondary antibodies that are Alexa Fluor® 488 conjugated (green).

**Figure 7 pone-0024075-g007:**
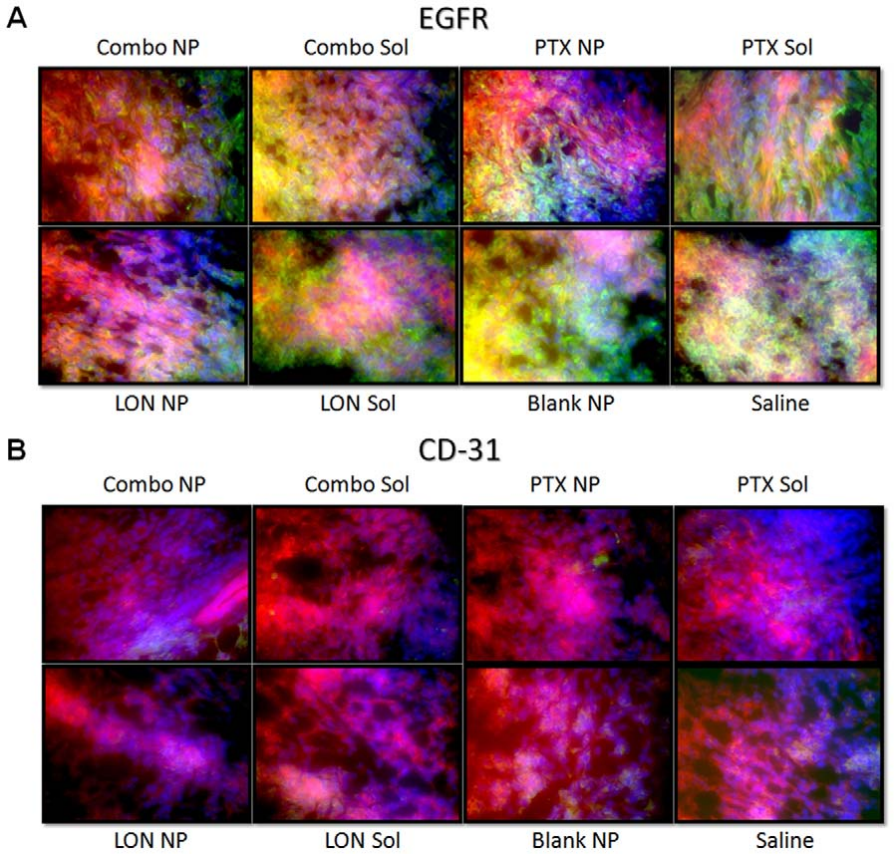
Immunohistochemistry of EGFR and CD-31. Tissue sections were probed with primary antibodies against the protein of interest, then labeled with Alexa Fluor® 488 conjugated secondary antibodies (green). F-actin was stained with Alexa Fluor® 568 phalloidin (red) and nuclei were stained with Hoechst 33342 (blue).

**Figure 8:**
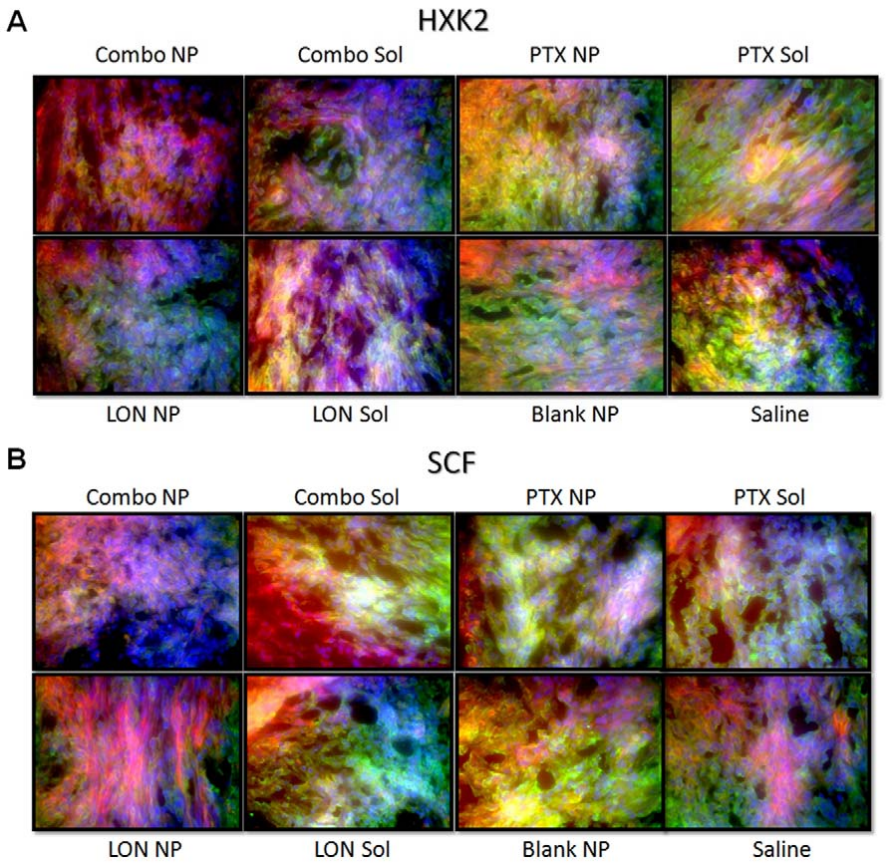
Immunohistochemistry of HXK2 and SCF. Tissue sections were probed with primary antibodies against the protein of interest, then labeled with Alexa Fluor® 488 conjugated secondary antibodies (green). F-actin was stained with Alexa Fluor® 568 phalloidin (red) and nuclei were stained with Hoechst 33342 (blue).

P-gp expression (**Figure 6**) was very high in the saline group, the blank NP group, and in the LON SOL group. There appeared to be a slight decrease in the LON NP group, the PTX SOL group, the PTX NP group and the combination SOL group. There was a more remarkable decrease in the combination NP group. It is possible that a month of treatment with combination therapy begins to reverse MDR when administered as SOL (slight decrease in P-gp), but more completely reverses MDR when administered as NP treatment (dramatic decrease).

HIF-1α (**Figure 6**) was very high in the saline group, blank NP treated group, PTX SOL group, and PTX NP group. In these sections with high expression, HIF-1α appears to be co-localized with both the cytoplasmic cell fraction (yellow and green) and with the nucleus of cells (white). LON SOL treatment and more remarkably, LON NP treatment decreased HIF-1α expression. As LON inhibits hexokinase 2 (and the Warburg effect), this decrease in HIF-1α expression may be due to the feedback loop between the glycolitic and HIF pathways. There is a further decrease in HIF-1α expression with the combination treatment. LON may decrease the Warburg effect, decrease the activity of the HIF pathway, and sensitize the tumor to the effects of PTX, leading to MDR reversal.

There was a very high level of EGFR (**Figure 7**) expression in all of the tissue sections; however the combination NP group appeared to have slightly lower levels relative to the other groups. This may indicate that the aggressiveness and overall character of the tumor has transformed. On the other hand, CD-31 (**Figure 7**) expression was low/non-detectable in all tumor sections. CD-31 was included as a marker for angiogenesis.

The expression of HXK2 (**Figure 8**) followed a similar pattern to HIF-1α expression. There was a high level of expression in the saline treated group, the blank NP group, the PTX SOL group, and the PTX NP group. There was a decrease in HXK2 expression in the LON SOL treated group and a further decrease in the LON NP treated group, the combination SOL group, and the combination NP group. LON NPs appear to have more of an effect on HXK2 expression than LON SOL, and this effect seems to be enhanced with combination therapy.

The last protein that was examined was SCF (**Figure 8**). There was a moderate level of SCF expression in the saline treated group; this expression appeared more co-localized with F-actin (yellow) in the blank NP treated group; and appeared more co-localized with cell nuclei (white) in the LON SOL group, the PTX SOL group, and the PTX NP group. There appeared to be both F-actin and nucleic co-localization in the combination SOL treated group while there was a marked decrease in expression in the LON NP group and a further decrease in the combination NP treated group.

Collectively, the saline and blank NP treated groups have higher expression levels of P-gp, HIF-1α, EGFR, and HXK2 relative to the other treatment groups. Treatment with combination NPs resulted in a decrease in expression of P-gp, HIF-1α, EGFR, HXK2 and SCF relative to the other groups. Although less remarkable, treatment with the combination SOL demonstrated a similar pattern. This indicates that combination treatment does indeed transform the innate character of the MDR tumors.

## Discussion

The complete *in vitro* characterization of this drug delivery system demonstrated the improved efficacy of combination PTX/LON therapy, EGFR binding of the NPs, and sustained drug release of the system [17]. We developed a novel, orthotopic model of MDR human breast cancer using hypoxic pre-conditioned cells to establish tumor xenografts in nude mice (submitted for publication and briefly described in this work). Using this *in vivo* model, evaluations of this EGFR-targeted NP system demonstrated that these particles had a superior pharmacokinetic profile (T_½_,AUC_0-∞_,AUMC_0-∞_, and MRT_0-∞_) relative to a comparable non-targeted NP system [18]. The current research demonstrates that treatment with EGFR-targeted LON/PTX NPs is more effective than combination SOL treatment (decreased tumor volume and decreased tumor weight). The increased efficacy of NP treatment may be due to the enhanced permeability and retention effect and active targeting. Although combination therapy with drug SOL was also effective relative to single agent treatment, the toxicity associated with SOL treatment was much higher compared to NP treatment. The combination NPs resulted in less of a decrease in body weight and more of a recovery in body weight, less LDH, less ALT, lower WBC counts, and higher platelet counts. The higher toxicity of SOL treatment relative to the NPs is most likely due to the effects of Cremophor® EL. The significance between the saline group and the PTX SOL group as well as the combination SOL group is most likely due to a synergist combination of the cytotoxicty of PTX and the toxicity of the Cremophor® EL SOL. NP treatment is a safer alternative to combination drug SOL. Qualitative analysis demonstrated that combination NP treatment resulted in a more dramatic decrease in tumor core and perimeter density relative to combination SOL. This change in density may be proportional to the therapeutic effect.

Qualitative IHC analysis of protein expression also demonstrated a more pronounced decrease in the expression of Pgp, HIF-1α, EGFR, HXK2, and SCF after treatment with combination NPs. SCF was included in the IHC analysis to examine if treatment had an effect on the expression of SCF and to examine if SCF is indeed a marker for MDR in this tumor model. It appears that two core concepts of cancer stem cells co-exist; there are cancer *initiating* stem cells that originate as stem cells, but transform into cancer causing cells and secondly, there are cancer *derived* stem cells which are cancer cells that develop stem-like properties, these cells are better known as MDR cells. In line with the concept that MDR cells can develop stem-like properties and be identified as cancer stem cells, different studies have shown that cell stressors such as hypoxia, which are efficient in inducing cancer aggression and MDR phenotypes, also induce stem-like properties in cancer cells such as the expression of stem cell factor (SCF) [3], [4], [9], [19], [20]. It may be that as the tumors become less hypoxic (LON NPs and combination NPs), SCF also decreases. The differential co-localization of SCF may be attributed to its role in many different signally pathways such as the c-kit pathway; also there is a soluble and transmembrane form of SCF. The different treatments may alter distinct signally pathways dominated by one of the SCF isoforms.

Overall, the IHC demonstrated that combination NP treatment appears to change the phenotype of the tumor, decreasing the MDR character of the xenografts (**Figure 1**). Collectively, the EGFR-targeted NPs were more effective in treating MDR than SOL and single agent treatments and less toxic than the SOL treatments. This nanocarrier system is a stepping stone on the road to personalized medicine.

### Concluding Remarks

Treating MDR with a cocktail of chemotherapeutic agents is a common clinical approach. However, as MDR is a dynamic disease state, many of the current drug combinations are rendered ineffective after perpetual use. As such, there is a demand for new drug combinations for treating MDR. There is also a clinical need to reduce the toxicity associated with these treatments as toxicity often demands dose reduction and/or an increase in the dosing interval which can aid the development of acquired MDR. Combination LON/PTX therapy using EGFR-targeted NPs represents a new approach for the treatment of MDR cancer; this approach addresses the clinical demand for new drug combinations and provides a solution to chemotherapy associated toxicity through the use of a nanocarrier system.

## Materials and Methods

### Polymer and Peptide Conjugation

This nanocarrier system has been completely characterized and described in our other works [17], [18]. The peptide GE11 was used to accomplish active targeting of the EGF receptor; this is an established EGFR ligand with the following sequence: YHWYGYTPQNVIGGGGC [21], [22]. Conjugation of GE11 to the PLGA-PEG construct was achieved using maleimide/cysteine linakge (the PEG residue has a terminal maleimide while the peptide has a terminal cysteine). GE11 was synthesized by Tufts University Core Facility, Boston, MA. To synthesize both the PLGA-PEG-peptide and PLGA-PEG constructs, 50∶50 poly(DL-lactide-*co*-glycolide) (PLGA) with an inherent viscosity of 0.15-0.25 (Durect Lactel® Adsorbable Polymers; Pelham, AL) was used; amine- poly(ethylene glycol) PEG-maleimide (MW 2000; JenKem Technology; Allen, TX) was used for the PLGA-PEG-peptide construct, while m-PEG-amine (MW 2000; LaysanBio; Arab, AL) was used to synthesize the PLGA-PEG construct. For complete details of the construct synthesis, please refer to our prior publication [17].

### Nanoparticle and Drug Solution Preparations

An established solvent displacement method was used to synthesize the NPs [23]. Poly(ɛ-caprolactone) (PCL; average MW 14.8 kDa; Polysciences, Inc., Warrington, PA) was used as the primary NP constituent. PCL, the PLGA-PEG-peptide construct, and the therapeutic agents were dissolved in 2 mL 50/50 acetonitrile/DMF then incubated in a 37°C water bath for 10 minutes. This SOL was then added dropwise to 20 mL distilled, deionized water while stirring, covered with aerated parafilm, allowed to stir overnight, centrifuged at 10,000 g for 30 minutes, and then resuspened in di water. The PLGA-PEG-peptide conjugate was added to the NP formulation at 20% w/w total polymer. An additional 10% w/w of PLGA-PEG conjugate was also added to ensure PEG modification and prevent clearance by the reticuloendothelial system. Combination NPs were synthesized with a 10∶1 molar ratio of LON to PTX. A Hitachi S-4800 microscope was used to obtain SEM images of the NPs.

Cremophor® EL (polyoxyethylated castor oil) was used to prepare drug SOL stocks. Each mL contained 527 mg of Cremophor® EL (BASF, Mount Olive, NJ, USA), 49.7% (v/v) dehydrated alcohol, USP (Thermo Fisher Scientific, Waltham, MA), 3 mg PTX, and 12 mg LON.

### Cell Culture and Hypoxia

The MDA-MB-231 cells were obtained from ATCC (Manassas, VA), they were incubated at 37°C and maintained in RPMI-1640 media (Mediatech, Inc; Manassas, VA) supplemented with 1% penicillin/streptomycin/amphotericin B mixture (Lonza; Walkersville, MD) and 10% fetal bovine serum (Gemini Bio-products; West Sacramento, CA). A low-oxygen gas (0.5% O_2_, 5% CO_2_, nitrogen balanced) was used to create hypoxic conditions; cell culture flasks were placed in a modular incubation chamber (Billups-Rothenberg, Inc.; Del Mar, CA), flushed with the gas for five minutes, and incubated at 37°C for five days.

### Animals and Orthotopic Model Development

The protocol for animal experiments described in this article was approved by Northeastern University's Institutional Animal Care and Use Committee (protocol#: 09-0724R). Female *nu/nu* mice purchased from Charles River Laboratories (Wilmington, MA) were housed in sterile cages on a 12∶12 light/dark cycle with ad libitum acess to food and water. Hypoxic pre-conditioned MDA-MB-231 cells were used to establish MDR tumor xenografts; mice were anesthetized with isoflurane and 2 million hypoxic MDA-MB-231 cells suspended in a 100 µl of a 50∶50 mix of matrigel and serum free medium was injected into the mammary fat pad of the mice using pre-chilled, sterile syringes with 27 gauge, ½’’ needles. Vernier calipers were used to measure tumor size every other day post-inoculation. Tumor volume (V) was calculated using the formula V  =  [length × (width)^2^]/2 where length is the longest diameter and width is the shortest diameter perpendicular to length.

### Animals, Treatment, and Tissue Preparation

When tumors reached a volume of 100 mm^3^, the mice were randomly selected for experimental treatment. Treatment was administered as a single tail vein injection; a 125 µL dose of 80 mg/kg LON and 20 mg/kg PTX. In addition to the eight treatment groups, a control group of mice without tumors were also included for the blood analysis. Four mice were included in each group.

At day zero (day of treatment initiation), day 14, and day 28 (day of animal sacrifice), 200 µL of blood was collected via retro-orbital bleeding. Mice were anesthetized by isoflurane inhalation and StatSpin® Microtubes (StatSpin, Inc., Norwood, MA) and capillaries were used for blood collection. Commercially available kits and their corresponding methods were used to measure plasma LDH and ALT; QuantiChromTM Lactate Dehydrogenase Kit (BioAssay Systems, Hayward, CA) and Liquid ALT (SGPT) Reagent Set (Pointe Scientific, Canton, MI).

After 4 weeks of treatment (day 28) animals were euthanized via isoflurane anesthesia overdose followed by carbon dioxide inhalation. After euthanasia, the tumor mass was collected and weighed, then prepared for IHC analysis.

### Immunohistochemistry of Tumors

Tumors were excised, embedded in tissue section medium (Richard-Allan Neg 50*, Thermo Scientific, Waltham, MA), flash frozen in liquid nitrogen, and stored at −80°C until use. Prior to cryo-sectioning, tumors were thawed to −20°C, then cut into 7 µm thick sections, mounted onto glass slides (SuperFrost Plus®, Thermo Scientific, Waltham, MA), outlined with an Aqua Hold Pap Pen (Scientific Device Laboratory, Des Plaines, IL), and air dried at room temperature. Sections were then fixed in ice-cold acetone for 10 minutes, air dried at room temperature for 1 hour, rinsed in two changes of cold PBS (5 minutes each), and incubated with 100 µl of IHC Select® Blocking Reagent (Chemicon, Billerica, MA) in a humidified chamber at 37°C for 30 minutes. The slides were rinsed in PBS and each section was incubated overnight at 4°C with 100 µl of primary antibody diluted in IHC Select® Antibody Diluent Solution (Chemicon, Billerica, MA). Slides were rinsed in two changes of PBST, each section was incubated with 100 µl of secondary antibody diluted in IHC Select® Antibody Diluent Solution at room temperature for 30 minutes, slides were washed in two changes of PBS then incubated with a SOL of Alexa Fluor® 568 phalloidin (to stain F-actin) and Hoechst 33342 (to stain nuclei) (Invitrogen; Carlsbad, CA) for 20 minutes, and then slides were rinsed with PBST and dehydrated in 95% ethanol for 2 minutes and 100% ethanol for two exchanges (3 minutes each). Tissue sections were mounted with Prolong Gold® Antifade reagent (Invitrogen; Carlsbad, CA). All primary antibodies were from Cell Signaling Technology (Danvers, MA) while the secondary antibodies were Alexa Fluor® 488 goat anti-mouse IgG (H+L) and Alexa Fluor® 488 goat anti-rabbit IgG (H+L) (Invitrogen; Carlsbad, CA). An Olympus IX51 Microscope was used to image the tissue sections.

### Hematoxylin and Eosin Staining of Tumor Sections

Tissue slices were prepared and fixed according to the methods described above. After fixing in acetone the slides were immersed in 100% ethanol for 5 minutes, rinsed with water for 5 minutes, and then incubated with hematoxylin for 10 minutes in a light protected container. Slides were then rinsed with water for 10 minutes, dipped into a jar of 0.1% HCl 3 times, and dipped into water 3-4 times. This was followed by dipping the slides in; 0.1% ammonium hydroxide 3 times, into water 4 times, and in eosin for 3 minutes. Slides were then dipped into ethanol with 0.1% acetic acid five times, two exchanges of 100% ethanol five times each, two changes of acetone five times each, and then two exchanges of xylene-substitute five times each. Two drops of mounting agent was applied to each tissue section and they were covered with glass coverslips.

### Statistical Analysis and Graphing

GraphPad Prism® Software was used for all graphs and statistical analysis. A two-way ANOVA was used to analyze the efficacy data.

## Supporting Information

Figure S1 **Toxicity analysis at day 0, day 14, and day 28**. Liver enzymes (LDH and ALT) and blood counts were used to assess toxicity. For all graphs, *p<0.05, **p<0.01, ***p<0.0001. (**A**) LDH. The asterisks above the bars indicate significance between Saline and the indicated group. Not shown: significance between the No Tumor group and all treatment groups at 14 days (*p*<0.01) and 28 days (*p*<0.05). (**B**) ALT. The asterisks directly above the error bars (with no brackets) indicate significance between Saline and the indicated group. Not shown: significance between the No Tumor group (as well as the Vehicle group) and all solution groups at 14 days (*p*<0.01 for No Tumor comparison, *p*<0.05 for Vehicle comparison). (**C**) White Blood Cell (WBC) Counts. The asterisks directly above the error bars (with no brackets) indicate significance between Saline (as well as Vehicle) and the indicated group. The asterisks directly below the No Tumor bars indicate significance between the No Tumor group and all treatment groups for each time point. (**D**) Platelet Counts. The asterisks directly above the error bars (with no brackets) indicate significance between the No Tumor group and the indicated group. The significance indicated by the brackets (for Saline v. each solution group) is the same for Vehicle v. solution groups.
(TIF)
