# Supplementary figures and images for: Therapeutic Efficacy and Safety of Paclitaxel/Lonidamine Loaded EGFR-Targeted Nanoparticles for the Treatment of Multi-Drug Resistant Cancer
Source: PLoS One. 2011 Sep 8;6(9):e24075. doi: 10.1371/journal.pone.0024075 (PMC3169576; doi:10.1371/journal.pone.0024075)

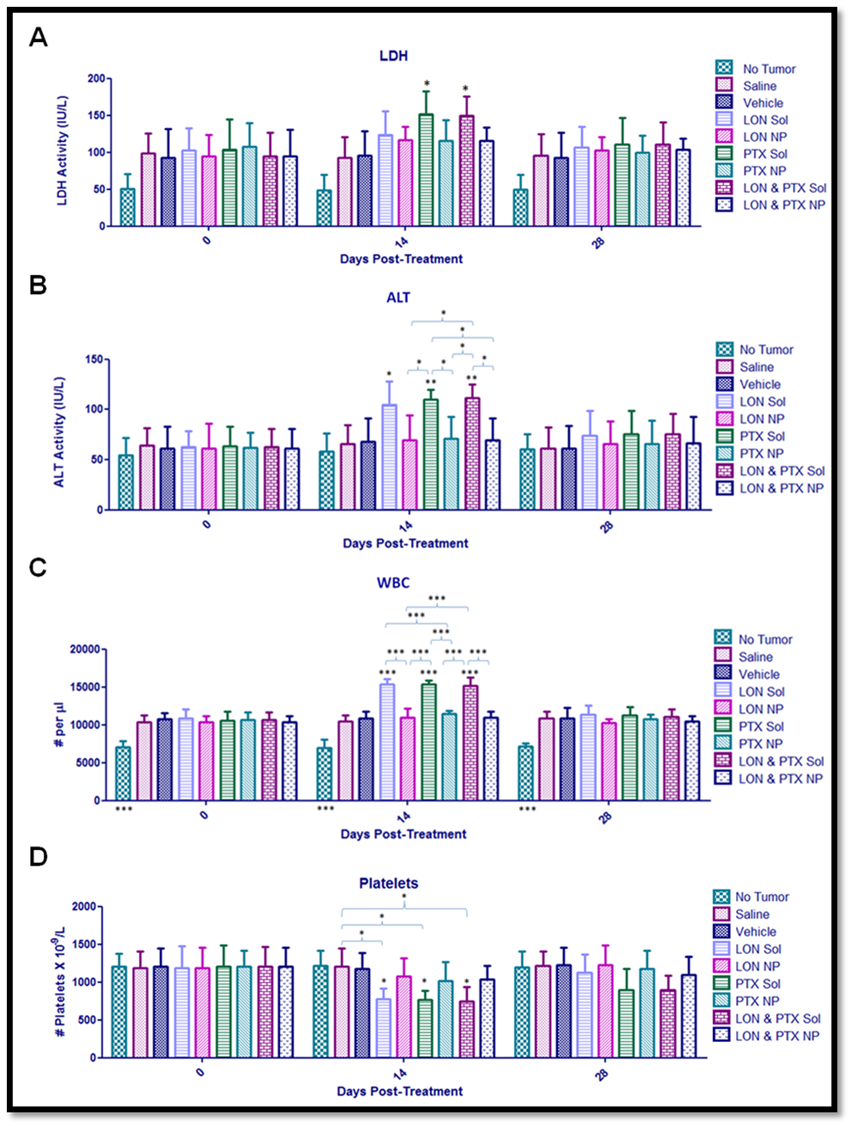

Supplement: Figure S1 — Toxicity analysis at day 0, day 14, and day 28. Liver enzymes (LDH and ALT) and blood counts were used to assess toxicity. For all graphs, *p<0.05, **p<0.01, ***p<0.0001. (A) LDH. The asterisks above the bars indicate significance between Saline and the indicated group. Not shown: significance between the No Tumor group and all treatment groups at 14 days (p<0.01) and 28 days (p<0.05). (B) ALT. The asterisks directly above the error bars (with no brackets) indicate significance between Saline and the indicated group. Not shown: significance between the No Tumor group (as well as the Vehicle group) and all solution groups at 14 days (p<0.01 for No Tumor comparison, p<0.05 for Vehicle comparison). (C) White Blood Cell (WBC) Counts. The asterisks directly above the error bars (with no brackets) indicate significance between Saline (as well as Vehicle) and the indicated group. The asterisks directly below the No Tumor bars indicate significance between the No Tumor group and all treatment groups for each time point. (D) Platelet Counts. The asterisks directly above the error bars (with no brackets) indicate significance between the No Tumor group and the indicated group. The significance indicated by the brackets (for Saline v. each solution group) is the same for Vehicle v. solution groups. (TIF) [file pone.0024075.s001.tif]
